# Rethinking depression diagnosis in ovarian cancer: The role of somatic symptoms

**DOI:** 10.1002/cncr.70344

**Published:** 2026-05-11

**Authors:** Rachel Telles, Premal H. Thaker, Michael J. Goodheart, Frank J. Penedo, Anil K. Sood, Susan K. Lutgendorf

**Affiliations:** ^1^ Department of Psychological and Brain Sciences University of Iowa Iowa City Iowa USA; ^2^ Division of Gynecologic Oncology, Department of Obstetrics and Gynecology Washington University School of Medicine and Siteman Cancer Center St. Louis Missouri USA; ^3^ Holden Comprehensive Cancer Center University of Iowa Iowa City Iowa USA; ^4^ Department of Obstetrics and Gynecology University of Iowa Iowa City Iowa USA; ^5^ Departments of Psychology and Medicine and Sylvester Comprehensive Cancer Center University of Miami Miami Florida USA; ^6^ Departments of Gynecologic Oncology and Cancer Biology and Center for RNA Interference and Noncoding RNA University of Texas MD Anderson Cancer Center Houston Texas USA

**Keywords:** assessment, depression, measurement, ovarian cancer survivors

## Abstract

**Background:**

A recent meta‐analysis reported that patients with ovarian cancer have a 3‐fold increase in the risk of diagnosis of depression compared to the general population. However, the role of disease‐related processes in depressive symptoms—particularly at diagnosis versus after treatment—remains unclear. This study aimed to examine the contribution of somatic symptoms to the assessment of depression severity in patients with ovarian cancer, both at diagnosis and 1 year later, compared to healthy controls.

**Methods:**

A total of 428 patients with ovarian cancer completed psychosocial assessments at 1–2 weeks before surgical intervention or initiation of neoadjuvant chemotherapy and at a 1‐year follow‐up visit. A comparison sample from the Midlife in the United States study was included. Item factor analysis was used to examine the functioning of somatic items in a common depression symptom index in both samples.

**Results:**

Somatic items demonstrated differential functioning between groups. Specifically, patients with ovarian cancer were more likely to endorse somatic symptoms at lower levels of depression as compared to healthy aging adults; they additionally required a lower level of depression to endorse somatic items as compared to nonsomatic items. These differences between patients with cancer and healthy aging adults were no longer present at 1 year postdiagnosis.

**Conclusions:**

These findings support the conclusion that somatic symptoms may disproportionately inflate depression scores among patients with ovarian cancer at diagnosis, which may potentially lead to misclassification or overestimation of depression severity. This highlights the need for refined measurement approaches that account for the somatic burden of cancer in assessing depression during active disease.

## Abstract 2

Patients with ovarian cancer endorse somatic symptoms of depression at a significantly lower severity of depression than the general population at time of diagnosis, which reflects possible overdiagnosis of depression as a result of cancer‐related symptom burden. Differences are no longer present at 1 year posttreatment, when somatic items may be more appropriate for depression assessment in this population.

## INTRODUCTION

Ovarian cancer is the most lethal gynecologic malignancy despite being only the third most commonly diagnosed worldwide, with a modest 10‐year survival rate of just 35%.^1^ Survival drops precipitously for women with advanced disease,^2^ which may explain why patients with ovarian cancer experience higher rates of psychological distress compared to other cancers with more favorable prognoses.^3^ Distinguishing normative emotional responses from clinical mood disorders such as major depressive disorder is particularly relevant,^4^ given reports linking depressive disorders to greater mortality.^5^, ^6^

Whereas lifetime prevalence of depression in the general population is estimated at 8.3%,^7^ meta‐analytic data suggest that more than 25% of patients with ovarian cancer meet the criteria for depressive syndromes before treatment, with some estimates as high as 83%–92%.^8^, ^9^, ^10^ Rates decline during and after treatment, which suggests a temporal association between disease trajectory and depressive symptoms.^10^ This pattern raises questions as to whether the high prevalence reflects clinical depression or is an adaptive response to illness.

Diagnosis is further complicated because somatic symptoms common in cancer—fatigue, appetite loss, and cognitive impairment—overlap with diagnostic criteria for depression, and may arise from cancer‐related inflammation and “sickness behavior.”^11^, ^12^, ^13^ Common depression scales, such as the Center for Epidemiologic Studies Depression Scale (CES‐D), may overestimate depression by conflating physical illness symptoms with psychological distress.^14^ These challenges are especially salient at diagnosis and early treatment, when physical symptoms peak.^13^ Previous studies, mostly in breast cancer populations and limited to single time points, show mixed findings.^15^, ^16^, ^17^ In ovarian cancer, first‐line treatment typically lasts approximately 6 months, with many patients achieving remission,^18^ which potentially reduces the confounding influence of somatic symptoms over time.

This study examined how somatic symptoms are reported relative to other depressive symptoms in patients with ovarian cancer at diagnosis and 1 year postdiagnosis compared to healthy controls. We hypothesized that at diagnosis, patients would report somatic symptoms more frequently than healthy controls and at a lower depression severity, and that somatic items would be endorsed at a lower severity than nonsomatic items. We further hypothesized that these differences would diminish at the 1‐year follow‐up, when disease processes no longer drive somatic symptoms.

## MATERIALS AND METHODS

This secondary analysis drew from two participant samples. The first sample included women diagnosed with ovarian cancer enrolled in a larger longitudinal study on biobehavioral factors in epithelial ovarian cancer progression. The second sample was a healthy aging cohort from the Midlife in the United States (MIDUS) study. Full methodological details, including survey procedures and supplemental measures, are available in Supporting Information S1.

### Ovarian cancer sample

Participants were recruited at their initial clinic visit for suspected ovarian cancer from three National Cancer Institute–designated cancer centers (University of Iowa, Washington University in St. Louis, and University of Miami). Exclusion criteria included age younger than 18 years, prior cancer, immunologically relevant comorbidities, recent corticosteroid use, inability to complete assessments, and diagnosis of benign disease, nonovarian or nonepithelial malignancies, or low malignant potential tumors.

All study procedures were institutional review board approved, and all took place after consent was obtained. For inclusion in this analysis, participants were required to have completed at least 50% of baseline surveys. The final sample included 428 women (mean age, 54.05 ± 11.53 years; 96% White).

### Healthy aging comparison sample

The comparison group comprised women from the MIDUS 2 Biomarker Project (2004–2006), originally a national longitudinal survey of 7000 adults. Inclusion in this analysis required participants to be female, to have completed at least 75% of the CES‐D, and to have no lifetime history of a cancer diagnosis. The final analytic sample included 713 women (mean age, 59.8 ± 11.63 years; 75.5% White; 95.9% non‐Hispanic). Details of the MIDUS study have been previously described.^19^

### Measures

#### Demographic and clinical characteristics

Participants with ovarian cancer self‐reported sociodemographics, including age, race, marital status, education, income, and employment. Clinical data—disease stage (I–IV), tumor grade, histology, chemotherapy history, and medication use—were extracted from medical records.

#### Psychological measurement of depression

Depressive symptoms were assessed with the 20‐item CES‐D, which measures symptom frequency over the past week (0 [rarely] to 3 [most of the time]).^20^ ^,^ ^21^ Items included both somatic (e.g., appetite loss and restless sleep) and nonsomatic (e.g., sadness and anhedonia) symptoms. The CES‐D was administered at baseline and at 1 year for patients with cancer; MIDUS participants completed the CES‐D at their biomarker visit. For this analysis, the items classified as somatic included items 2 (“I did not feel like eating; my appetite was poor”), 5 (“I had trouble keeping my mind on what I was doing”), 7 (“I felt that everything I did was an effort”), 11 (“My sleep was restless”), 13 (“I talked less than usual”), and 20 (“I could not get ‘going’”), consistent with prior factor analysis.^20^, ^22^

Presurgery, patients with cancer were administered the Structured Clinical Interview for Depression (SCID) of the *Diagnostic and Statistical Manual of Mental Disorders* (Fourth Edition) (*DSM‐IV*) major depressive disorder screening question, “Have you ever had a period in your life when you felt depressed most of the day, every day, for at least 2 weeks?”^23^ Patients with positive responses were subsequently assessed with the SCID modules for mood episodes, major depression, dysthymic disorder, and anxiety disorders. The SCID‐IV, a structured diagnostic interview based on the criteria for depression in the *DSM‐IV*,^23^ was administered by a trained member of the study staff to patients with ovarian cancer within several weeks postsurgery. The SCID is the most widely used diagnostic tool for assessing *DSM*‐defined depression, and is considered the gold standard for the assessment of depression.^24^, ^25^, ^26^ To minimize burden, patients who answered “no” to the screening question were not subsequently assessed with the SCID. Of the 428 women, 95 answered “yes” to the screening question, and completed the SCID.

### Statistical analysis

#### Preliminary and descriptive analyses

Data were analyzed with SPSS, version 29,^27^ and R, version 4.4.2.^28^ Distributions were checked for normality and outliers.

#### Primary analyses

Further details on power analysis and model specification may be found in Supporting Information S1. The prevalence of depressive disorders in patients with ovarian cancer was assessed with CES‐D cutoff scores of ≥16 (standard) and ≥27 (more specific for clinical risk) alongside SCID‐IV data.^29^, ^30^

The current analyses were performed with item factor analysis (IFA), which analyzes the underlying structure of survey responses. Depression screener questionnaires assume that all items are assessing one construct (depression) but responses are often driven by multiple constructs (e.g., fatigue, depression, anxiety, and illness). Additionally, different items may assess different levels of depression and function differently in the amount of information they offer about depressive symptoms. IFA examines shared variance across items, and helps to identify how closely those items are associated with depression (measured by factor loadings) and how depressed a patient must be to endorse the item (measured with item thresholds). When considering depression as a spectrum ranging from “not depressed” to “severely depressed,” it is important to know whether a certain score indicates the same level of depression across different groups (also known as “measurement invariance”). Measurement noninvariance testing assessed differences in somatic item thresholds between patients with ovarian cancer and controls, and between somatic and nonsomatic items within the cancer group. IFA was conducted in R.^31^ Two models were estimated: one combining both groups, and one limited to patients with ovarian cancer. The first model tested differences between patients with cancer and healthy comparisons on the level of depression required to endorse somatic items, both at time of diagnosis and at 1 year thereafter. The second model compared the level of depression required to endorse somatic items as compared to nonsomatic items only by patients with cancer. In other words, the model investigated whether patients with cancer would need to have more severe depression to endorse nonsomatic items. Model fit was evaluated with the root mean square error of approximation (RMSEA) (<0.06, good; <0.08, acceptable) and comparative fit index (CFI) (>0.95, good; >0.90, acceptable).

After the fitting of these models, item thresholds were averaged for each item (where each item initially had three thresholds) and then averaged across items for somatic and nonsomatic items accordingly.^20^, ^32^ Differences in average thresholds were tested across groups and item types. A follow‐up model allowed factor loadings to vary by group to assess structural differences. All threshold analyses were repeated at 1 year.

## RESULTS

### Clinical and demographic characteristics

Table 1 presents sample characteristics. The ovarian cancer sample (*N* = 428) was predominantly White (96%) and middle aged (mean age, 54.05 ± 11.53 years; range, 27–89 years), and most patients had advanced‐stage (72%) and high‐grade (84.3%) disease. The healthy comparison sample from the MIDUS study (*N* = 713) was also primarily White (75.5%), non‐Hispanic (95.9%), and middle aged (mean age, 59.8 ± 11.63 years; range, 34–84 years). On average, patients with cancer endorsed every somatic item at higher levels than the healthy comparison group (Table 2), and endorsement of somatic items as occurring much of the time or more was higher across all items for patients with cancer (Table 3).

**TABLE 1:** Demographic and clinical characteristics of patients with ovarian cancer.

Measure	Patients with ovarian cancer (*N* = 428)	Healthy comparison sample (*N* = 713)	*p*
Age at study entry, years			
Mean (SD)	54.05 (11.53)	59.8 (11.63)	<.001
Race, No. (%)			<.001
American Indian/Alaskan Native	2 (0.5)	9 (1.3)	
Asian	3 (0.7)	1 (0.1)	
Black	11 (2.6)	145 (20.3)	
White	411 (96.0)	538 (75.5)	
Unknown	1 (0.2)	4 (0.6)	
Other		16 (2.2)	
Ethnicity, No. (%)			.39
Hispanic	17 (4.0)	26 (3.7)	
Non‐Hispanic	411 (96.0)	684 (95.9)	
Cancer stage, No. (%)			
I	85 (19.9)		
II	30 (7.0)		
III	266 (62.1)		
IV	42 (9.8)		
Unknown	5 (1.2)		
Cancer grade, No. (%)			
Low	61 (14.3)		
High	361 (84.3)		
Unknown	6 (1.4)		
Medical morbidities, No. (%)			
Autoimmune disorder		10 (1.4)	
Hypertension		144 (20.2)	
Diabetes/hyperglycemia		44 (6.2)	
Reported no. of chronic conditions, mean (SD)		2.62 (2.38)	

**TABLE 2:** Average scores on somatic items by group.

CES‐D item	Patients with cancer, mean ± SD	Healthy comparisons, mean ± SD	*p*
2: “I did not feel like eating; my appetite was poor”	1.29 ± 1.10	0.24 ± 0.56	<.001
5: “I had trouble keeping my mind on what I was doing”	1.04 ± 0.91	0.60 ± 0.78	<.001
7: “I felt that everything I did was an effort”	1.04 ± 0.98	0.53 ± 0.83	<.001
11: “My sleep was restless”	1.36 ± 1.03	0.99 ± 0.98	<.001
13: “I talked less than usual”	0.83 ± 0.86	0.52 ± 0.79	<.001
20: “I could not get ‘going’”	0.91 ± 0.91	0.53 ± 0.77	<.001

*Note:* Scores reflect those of patients with cancer at time of diagnosis.

Abbreviation: CES‐D, Center for Epidemiologic Studies Depression Scale.

**TABLE 3:** Prevalence of somatic item endorsement “much of the time” or more frequently.

CES‐D item	Patients with cancer, %	Healthy comparisons, %
2 (appetite)	36.2	4.8
5 (concentration)	24.3	13.5
7 (effort)	26.9	12.5
11 (sleep)	38.1	27.6
13 (talk)	19.4	13.0
20 (motivation)	22.0	11.2

Abbreviation: CES‐D, Center for Epidemiologic Studies Depression Scale.

### Somatic item performance in an IFA model: Thresholds

Average depressive symptoms as assessed by the CES‐D were significantly higher for patients with ovarian cancer at time of diagnosis (mean, 16.72 ± 9.92) than for the healthy comparison group (mean, 9.01 ± 8.40; *t*(626.75), 12.58; *p* < .001). A larger proportion of patients with ovarian cancer (*n* = 176; 41.1%) than healthy controls (*n* = 120; 16.8%) met the screening threshold denoting an elevated risk for depressive disorder (a score of 16 on the CES‐D). With the aforementioned higher cutoff of 27, a smaller proportion of both groups met the criteria; however, patients with ovarian cancer (*n* = 66; 15.4%) still met the criteria at a higher rate than the healthy comparisons (*n* = 34; 4.8%). In contrast, when the SCID‐IV was administered to patients with ovarian cancer, only a small percentage met the criteria for current major depressive disorder (*n* = 21; 4.9%) or dysthymia (currently called “persistent depressive disorder”; *n* = 8; 1.9%). Two IFA models for the CES‐D were fitted. One was fit to the full sample, which included both patients with ovarian cancer and healthy MIDUS comparisons; the second was fit only to patients with ovarian cancer. In both models, factor loadings were constrained, whereas thresholds were permitted to vary between groups. Item threshold parameters for each group from the first model are presented in Table S1. Model fit statistics for both models are presented in Table 4. Taken together, the model fit was acceptable for both models, although it was slightly better for the model that included only patients with cancer. Given the acceptable fit of both models, this allowed us to proceed in comparing threshold values.

**TABLE 4:** Model fit statistics.

Model	*χ* ^2^ value	*χ* ^2^ *df*	*χ* ^2^ *p*	CFI	RMSEA estimate	RMSEA 90% CI
Threshold IFA models						
Combined	1249.40	360	<.001	0.947	0.067	0.063–0.071
Cancer only	424.12	160	<.001	0.954	0.064	0.057–0.072
Factor loading IFA model						
Combined	1812.42	340	<.001	0.912	0.088	0.084–0.092
Threshold IFA models at 1 year						
Combined	841.38	360	<.001	0.972	0.052	0.048–0.057
Cancer only	263.99	160	<.001	0.985	0.049	0.038–0.059

*Note:* Scaled values are provided.

Abbreviations: CFI, comparative fit index; IFA, item factor analysis; RMSEA, root mean square error of approximation.

There was a significant difference in the average threshold for somatic items between the two groups (diff, 0.789; *p* < .001), which represented a significantly higher threshold for somatic items in the healthy comparison group. In this context, this indicates that patients with cancer reported somatic symptoms at a lower depression severity than the healthy controls.

The second model evaluated patients with cancer alone. There was a significant difference in the average threshold for somatic items compared to nonsomatic items (diff, −0.637; *p* < .001), which indicates a significantly lower threshold for somatic items in the group of patients with cancer. In this context, this indicates that patients with cancer reported somatic symptoms at a lower depression severity than where they reported nonsomatic items.

### Somatic item performance: Factor loadings

A secondary analysis was explored to evaluate the clinical utility of somatic items. Whereas the differences in thresholds suggest a need for caution in assuming a depressive disorder because of high sum scores reflecting largely somatic items, differences in factor loadings would suggest that the somatic items were not reflective of depression at all, and were instead measuring something else (e.g., disease). In this new model, both thresholds and factor loadings were unconstrained across groups. Model fit statistics for this model may be found in Table 4, and demonstrate an acceptable fit (although with an RMSEA slightly higher than generally accepted guidelines, which may potentially be due to the unconstrained nature of the model).

There was no significant difference in average factor loading for somatic items between the two groups (diff, −0.058; *p* = .269), which means that items were equally related to the overall measure in both groups. However, there was a significant difference in average factor loadings between somatic and nonsomatic items in patients with cancer (diff, 0.228; *p* < .001), such that nonsomatic items had stronger loadings. This indicated that the nonsomatic symptoms had a stronger association with depression than the somatic symptoms, which suggests that somatic symptoms are less central to depression in patients with cancer at time of diagnosis.

### Somatic item performance: Thresholds at 1 year

Two final models were fit mirroring the first model but with data for patients with ovarian cancer at 1 year postdiagnosis to determine whether the somatic thresholds had changed after the primary treatment. This sample was necessarily smaller, given that some patients (*n* = 38) had died and some patients (*n* = 112) had moved or did not respond to attempts to contact them. Baseline depressive symptoms did not vary among patients who died, those lost to attrition, and those who remained in the study (*p* = .103). The stage of disease at diagnosis also did not vary among the three groups (*p* = .142). The final sample at 1 year consisted of 273 patients.

The same two models were fit as in the prior analysis at baseline. Item threshold parameters for each group from the first model are presented in Table S1. Model fit statistics for both of these models may be found in Table 4. Taken together, model fit was acceptable for both models.

There was no significant difference in the average threshold for somatic items between patients with cancer at 1 year postdiagnosis and healthy adults (diff, 0.106; *p* = .106). In this context, this indicates that patients with cancer now endorsed somatic items at a similar depression severity as the healthy comparison group, which suggests that high levels of endorsement of somatic symptoms of depression are less prevalent 1 year after diagnosis.

The second model evaluated patients with cancer alone at 1 year postdiagnosis. There was a significant difference in the average threshold for somatic items compared to nonsomatic items (diff, −0.668; *p* < .001), which indicates a significantly lower threshold for somatic items in the group of patients with cancer. In this context, this indicates that patients with cancer still endorsed somatic items at a lesser depression severity than nonsomatic items, even at 1 year postdiagnosis.

## DISCUSSION

Previous research suggests that somatic items on depression scales measure depression similarly to nonsomatic items in cancer populations and do not require modification.^17^ However, in this homogeneous sample of women with ovarian cancer, we observed notable differences between patients and healthy controls. Somatic items were endorsed more often and as occurring more frequently by patients with ovarian cancer. Patients endorsed somatic items at a lower depression severity than nonsomatic items, whereas healthy individuals required a higher depression severity to report somatic symptoms. These between‐group differences disappeared 1 year postdiagnosis, although within‐group differences in endorsement remained. Factor loadings did not differ, which indicated that somatic items continued to reflect overall depression in both groups.

These findings contrast with studies focusing on heterogeneous cancer samples dominated by patients with breast cancer, who generally show lower symptom burden (although notably in a sample with higher depression rates).^15^ Ovarian cancer, often diagnosed at an advanced stage, presents with substantial pretreatment symptom burden—including fatigue, appetite loss, pain, and sleep disturbance—that can mimic depressive symptoms.^3^, ^33^, ^34^ This likely contributes to early somatic symptom endorsement unrelated to depression. Depression measures used in oncology populations have long been criticized for only moderate acceptability in nonpalliative settings.^35^ Although at least one study reports acceptable performance of somatic items,^16^ such studies rarely use a gold‐standard diagnostic method.

Calls for alternative assessments, such as use of the Endicott substitutive criteria to replace somatic items,^36^ have been made to improve diagnostic accuracy.^37^ However, such recommendations rarely account for cancer site–specific differences. Our findings underscore the need to examine depression assessment tools in homogeneous cancer populations, especially those such as ovarian cancer, where advanced disease and high symptom burden are typical.^38^, ^39^, ^40^

Accurate depression diagnosis at cancer diagnosis is critical, given that untreated depressive disorders are associated with greater mortality^5^, ^6^ and poorer adherence to cancer treatment.^41^ Given our findings, it is important to differentiate disease‐related somatic symptoms from depressive disorders. Although depressive symptoms often decline after diagnosis, a high‐risk subgroup maintains or develops clinically significant symptoms.^42^, ^43^ Even if measures such as the CES‐D are imperfect early in the cancer trajectory, they remain clinically valuable. Early intervention for major depression can reduce mortality risk by 45%.^44^ Understanding whether risk reflects true depression pathology or indirect measurement of disease severity is essential for targeted intervention.

One potential explanation for differences in symptom patterns is that depression in cancer may represent a partially distinct syndrome. Prior work examined cytokine‐associated depression, characterized by neurovegetative symptoms, which may differ from classical major depressive disorder.^45^, ^46^, ^47^ Future work should explore inflammatory markers and their association with depressive symptoms in ovarian cancer. Depression and physical symptom burden also share a bidirectional relationship; somatic symptoms may amplify depressive symptoms and vice versa, and thereby complicate the interpretation of temporality. This mirrors the palliative care concept of “total pain.”^48^

By 1 year postdiagnosis, patients with ovarian cancer endorsed somatic items at levels comparable to healthy controls, which suggests that acute disease processes drive early somatic symptoms. Depression assessment at diagnosis therefore requires a more nuanced interpretation to avoid overdiagnosis. After treatment, depression measures including somatic items may be more appropriate. Clinicians should be educated on symptom patterns, prioritize nonsomatic items (e.g., sadness and despair) in the early stages of cancer diagnosis and treatment, and provide psychoeducation on how symptoms may evolve. Patients may benefit from a multifaceted approach addressing both depressive and somatic symptoms. Research should consider the phase of diagnosis and treatment when selecting measures; the use of multiple tools may capture somatic symptom changes more accurately over time.

### Limitations

The current study has some limitations that must be considered when interpreting the results. The study sample was predominantly White and non‐Hispanic, which limits generalizability to more diverse populations. This is particularly relevant given the significant racial disparities in ovarian cancer outcomes; Black women face notably worse survival rates despite lower incidence.^49^ Additionally, the reliance on self‐report measures introduces possible bias (e.g., social desirability). Furthermore, self‐report measures of depression such as the CES‐D have low positive predictive value by often reflecting generalized distress rather than a true depressive disorder. It is also noteworthy that the CES‐D is more commonly used in research settings and not often used clinically; these results may not generalize to clinical settings but have important implications for how research on depression in this population may improve measurement moving forward. Although assessment was supplemented with a gold‐standard clinical interview (SCID), SCID‐determined depression rates were low; whether this reflects true prevalence or measurement limitations warrants further study, particularly in high‐risk or clinically depressed cancer samples. Comparison with a large community sample strengthened the study but the samples were not matched and came from different studies with differing sociodemographic characteristics and procedures, which potentially influences generalizability.

### Clinical implications

This study used a rigorous method to examine depression measurement in ovarian cancer and how item performance changes over time. Somatic symptoms were endorsed at lower levels of depression than nonsomatic ones, particularly before treatment. Structural validity remained intact, and group differences resolved after treatment. Psychosocial interventions should recognize that symptom burden and functional impairment may differ across cancers. Although much of the extant literature has focused on patients with breast cancer, because of differences in the somatic burden of disease these findings may not generalize to other patient populations, and should be interpreted with caution outside of breast cancer.

Future research should refine depression assessment during active cancer by emphasizing patient education, clinician–patient communication, and targeted management of physical symptoms. Incorporating screening measures that emphasize cognitive and emotional symptoms early in diagnosis alongside inventories that include somatic items may improve diagnostic accuracy.

## AUTHOR CONTRIBUTIONS

**Rachel Telles**: Conceptualization; formal analysis; methodology; writing–original draft; and writing–review and editing. **Premal H. Thaker**: Resources; investigation; supervision; and writing–review and editing. **Michael J. Goodheart**: Writing–review and editing and resources. **Frank J. Penedo**: Investigation and writing–review and editing. **Anil K. Sood**: Methodology; writing–original draft; and writing–review and editing. **Susan K. Lutgendorf**: Conceptualization; funding acquisition; methodology; investigation; data curation; writing–review and editing; and writing–original draft.

## CONFLICT OF INTEREST STATEMENT

Premal H. Thaker has received research funding from Merck and GlaxoSmithKline, has served as a consultant for Imunon, is a shareholder of Imunon, and has served on advisory boards for Iovance, AstraZeneca, GlaxoSmithKline, Zentalis, Novocure, Seagen, Immunogen/AbbVie, Caris, Merck, Imunon, Verastem, Mural Oncology, BioNTech, Corcept, Genmab, Genelux, Mersana, Aadi Bioscience, and Incyte. Anil K. Sood has served as a consultant for Kaida, Corcept, Onxeo, and Foundation Medicine, received research funding from Pfizer, and served on advisory boards for Mural Oncology and Advenchen. Susan K. Lutgendorf is a shareholder of AbbVie. The other authors declare no conflicts of interest.

## Supporting information

Supporting Information S1

## Data Availability

The data that support the findings of this study are available on request from the corresponding author. The data are not publicly available because of privacy or ethical restrictions.
