# Supplementary material for: Rethinking depression diagnosis in ovarian cancer: The role of somatic symptoms
Source: Cancer. 2026 May 11;132:e70344. doi: 10.1002/cncr.70344 (PMC13158647; doi:10.1002/cncr.70344)
Supplement: Supplementary file 1 — Supporting Information S1 [file CNCR-132-e70344-s001.docx]

Supplement

**S1 Methods**

**S1.1 Survey Procedures**

**S1.1.2 Ovarian Cancer Sample**

Baseline psychosocial packets were completed 1-2 weeks prior to initiation of treatment (surgery or neoadjuvant chemotherapy) following study enrollment. Structured clinical interviews were administered either in person or over the phone. Self-report measures were repeated at 6-month (T2) and 1-year follow-up (T3) visits.

**S1.3 Statistical Analysis**

**S1.3.1 Power**

Utilizing Mplus version 8.11 (Muthén & Muthén, 1998), Monte Carlo simulations estimated that the sample size utilized here is able to detect a threshold difference of 0.25 between groups at a power of 80% and an alpha of 0.05. At a difference level of 0.1, the model was well-powered to detect within-group differences and had 74% power to detect between-group differences.

**S1.3.2 Primary Analyses**

Depressive disorder was operationalized as screening positive for Major Depressive Disorder or Dysthymic Disorder (the SCID-IV diagnosis equivalent to Persistent Depressive Disorder in DSM-V).

WLSMV estimation and the THETA parameterization were used to estimate all models; WLSMV was selected as it is more well-suited to analyses of polytomous ordered data than the ML estimator. Model fit statistics describe the fit of the item factor model to the correlation matrix among the items for each sample tested (i.e., the combined sample and the ovarian cancer patients alone).

This analysis was not intended to test full measurement non-invariance, and as such, the multiple-step process required to test each element of non-invariance (including configural invariance, scalar invariance, and invariance of residuals) was not employed here. The only parameters of interest were the difficulty, or threshold, parameter and the factor loading; for this reason, the model was initially tested with the same factor structure and with factor loadings constrained to be equal across groups. Polytomous thresholds for each item were permitted to vary across groups to test for significant differences in threshold levels. Once threshold differences were identified, factor loadings were freed and permitted to vary to examine potential differences.

Table S1. Item Threshold Parameters for IFA Model 1 of CES-D by Sample.

| **CES-D Item** | **Ovarian Cancer Patient Sample (N = 428)** | **MIDUS Sample (N = 713)** |
| --- | --- | --- |
| **1 “I was bothered by things that don’t usually bother me.”** |  |  |
| **THRESHOLD 1** | -0.063 | 0.569 |
| **THRESHOLD 2** | 1.189 | 1.894 |
| **THRESHOLD 3** | 1.951 | 2.965 |
| **2 “I did not feel like eating; my appetite was poor.”** |  |  |
| **THRESHOLD 1** | **-0.606** | **1.042** |
| **THRESHOLD 2** | **0.333** | **1.958** |
| **THRESHOLD 3** | **0.977** | **2.887** |
| **3 “I felt that I could not shake off the blues even with help from my family and friends.”** |  |  |
| **THRESHOLD 1** | 0.361 | 1.715 |
| **THRESHOLD 2** | 2.098 | 2.995 |
| **THRESHOLD 3** | 3.161 | 4.257 |
| **4 “I felt that I was just as good as other people.” (reverse-scored)** |  |  |
| **THRESHOLD 1** | -0.920 | 0.402 |
| **THRESHOLD 2** | -0.026 | 1.150 |
| **THRESHOLD 3** | 0.830 | 1.786 |
| **5 “I had trouble keeping my mind on what I was doing.”** |  |  |
| **THRESHOLD 1** | **-0.576** | **0.177** |
| **THRESHOLD 2** | **0.749** | **1.289** |
| **THRESHOLD 3** | **1.605** | **2.317** |
| **6 “I felt depressed.”** |  |  |
| **THRESHOLD 1** | 0.024 | 1.222 |
| **THRESHOLD 2** | 1.934 | 2.545 |
| **THRESHOLD 3** | 3.019 | 3.787 |
| **7 “I felt that everything I did was an effort.”** |  |  |
| **THRESHOLD 1** | **-0.499** | **0.498** |
| **THRESHOLD 2** | **0.754** | **1.548** |
| **THRESHOLD 3** | **1.690** | **2.285** |
| **8 “I felt hopeful about the future.”** |  |  |
| **THRESHOLD 1** | -0.379 | -0.150 |
| **THRESHOLD 2** | 0.777 | 0.912 |
| **THRESHOLD 3** | 1.931 | 1.544 |
| **9 “I thought my life had been a failure.”** |  |  |
| **THRESHOLD 1** | 1.891 | 1.791 |
| **THRESHOLD 2** | 2.828 | 2.796 |
| **THRESHOLD 3** | 3.632 | 3.731 |
| **10 “I felt fearful.”** |  |  |
| **THRESHOLD 1** | -0.665 | 1.143 |
| **THRESHOLD 2** | 0.656 | 2.358 |
| **THRESHOLD 3** | 1.582 | 3.574 |
| **11 “My sleep was restless.”** |  |  |
| **THRESHOLD 1** | **-0.818** | **-0.320** |
| **THRESHOLD 2** | **0.262** | **0.657** |
| **THRESHOLD 3** | **1.025** | **1.470** |
| **12 “I was happy.” (reverse-scored)** |  |  |
| **THRESHOLD 1** | -0.758 | 0.105 |
| **THRESHOLD 2** | 0.816 | 1.796 |
| **THRESHOLD 3** | 2.232 | 3.081 |
| **13 “I talked less than usual.”** |  |  |
| **THRESHOLD 1** | **-0.232** | **0.417** |
| **THRESHOLD 2** | **0.971** | **1.352** |
| **THRESHOLD 3** | **2.039** | **2.303** |
| **14 “I felt lonely.”** |  |  |
| **THRESHOLD 1** | 0.523 | 0.821 |
| **THRESHOLD 2** | 1.770 | 1.855 |
| **THRESHOLD 3** | 2.643 | 2.611 |
| **15 “People were unfriendly.”** |  |  |
| **THRESHOLD 1** | 1.546 | 1.135 |
| **THRESHOLD 2** | 2.010 | 2.155 |
| **THRESHOLD 3** | 2.340 | 2.867 |
| **16 “I enjoyed life.”** |  |  |
| **THRESHOLD 1** | -0.075 | 0.456 |
| **THRESHOLD 2** | 1.097 | 1.785 |
| **THRESHOLD 3** | 2.204 | 2.918 |
| **17 “I had crying spells.”** |  |  |
| **THRESHOLD 1** | 0.073 | 1.572 |
| **THRESHOLD 2** | 1.337 | 2.429 |
| **THRESHOLD 3** | 2.284 | 3.201 |
| **18 “I felt sad.”** |  |  |
| **THRESHOLD 1** | -0.764 | 0.545 |
| **THRESHOLD 2** | 1.268 | 2.258 |
| **THRESHOLD 3** | 2.545 | 3.311 |
| **19 “I felt that people disliked me.”** |  |  |
| **THRESHOLD 1** | 1.802 | 1.163 |
| **THRESHOLD 2** | 2.573 | 2.314 |
| **THRESHOLD 3** | 2.915 | 2.922 |
| **20 “I could not get ‘going’.”** |  |  |
| **THRESHOLD 1** | **-0.360** | **0.400** |
| **THRESHOLD 2** | **0.984** | **1.653** |
| **THRESHOLD 3** | **2.040** | **2.604** |

Note: Values represent threshold values, which may be interpreted as relative level of depression required to endorse the item at each level (higher numbers represent higher severity of depression, with 0 interpretable as the average level of depression across the sample). Each item has three thresholds, representing the level of depression required to endorse “Some or a little of the time (1-2 days)”, “Occasionally or a moderate amount of the time (3-4 days)”, and “Most or all of the time (5-7 days)” respectively. Values are relative in nature and should only be interpreted in relation to each other. Bolded items represent somatic symptoms.Table S2. Descriptive Statistics by Missingness

| **Variable** | **Patients with Data at 1 Year (N = 273)** | **Patients Deceased at 1 Year (N = 38)** | **Patients Lost to Follow Up (N = 112)** | ***p* value** |
| --- | --- | --- | --- | --- |
| **Baseline**  **CES-D Score** (mean ± SD) | 15.85 ± 9.30 | 18.86 ± 10.27 | 17.63 ± 10.90 | .103 |
| **Stage**  I  II  III  IV | 61  23  165  24 | 2  2  28  6 | 22  5  73  12 | .142 |

Table S3. Item Threshold Parameters for IFA Model 3 of CES-D by Sample at 1 Year.

| **CES-D Item** | **Ovarian Cancer Patient Sample (N = 428)** | **MIDUS Sample (N = 713)** |
| --- | --- | --- |
| **1 “I was bothered by things that don’t usually bother me.”** |  |  |
| **THRESHOLD 1** | 0.306 | 0.594 |
| **THRESHOLD 2** | 1.849 | 1.978 |
| **THRESHOLD 3** | 2.709 | 3.096 |
| **2 “I did not feel like eating; my appetite was poor.”** |  |  |
| **THRESHOLD 1** | **1.033** | **1.094** |
| **THRESHOLD 2** | **1.974** | **2.055** |
| **THRESHOLD 3** | **2.828** | **3.030** |
| **3 “I felt that I could not shake off the blues even with help from my family and friends.”** |  |  |
| **THRESHOLD 1** | 1.038 | 1.856 |
| **THRESHOLD 2** | 2.837 | 3.239 |
| **THRESHOLD 3** | 4.101 | 4.605 |
| **4 “I felt that I was just as good as other people.” (reverse-scored)** |  |  |
| **THRESHOLD 1** | -0.045 | 0.424 |
| **THRESHOLD 2** | 0.745 | 1.214 |
| **THRESHOLD 3** | 1.578 | 1.886 |
| **5 “I had trouble keeping my mind on what I was doing.”** |  |  |
| **THRESHOLD 1** | **-0.055** | **0.181** |
| **THRESHOLD 2** | **1.295** | **1.317** |
| **THRESHOLD 3** | **1.953** | **2.367** |
| **6 “I felt depressed.”** |  |  |
| **THRESHOLD 1** | 0.705 | 1.473 |
| **THRESHOLD 2** | 2.839 | 3.069 |
| **THRESHOLD 3** | 4.082 | 4.565 |
| **7 “I felt that everything I did was an effort.”** |  |  |
| **THRESHOLD 1** | **0.038** | **0.512** |
| **THRESHOLD 2** | **1.390** | **1.591** |
| **THRESHOLD 3** | **2.482** | **2.349** |
| **8 “I felt hopeful about the future.”** |  |  |
| **THRESHOLD 1** | 0.099 | -0.151 |
| **THRESHOLD 2** | 1.108 | 0.915 |
| **THRESHOLD 3** | 2.111 | 1.549 |
| **9 “I thought my life had been a failure.”** |  |  |
| **THRESHOLD 1** | 1.714 | 1.840 |
| **THRESHOLD 2** | 2.952 | 2.873 |
| **THRESHOLD 3** | 3.677 | 3.832 |
| **10 “I felt fearful.”** |  |  |
| **THRESHOLD 1** | 0.509 | 1.152 |
| **THRESHOLD 2** | 1.891 | 2.378 |
| **THRESHOLD 3** | 2.839 | 3.604 |
| **11 “My sleep was restless.”** |  |  |
| **THRESHOLD 1** | **-0.538** | **-0.326** |
| **THRESHOLD 2** | **0.813** | **0.670** |
| **THRESHOLD 3** | **1.546** | **1.498** |
| **12 “I was happy.” (reverse-scored)** |  |  |
| **THRESHOLD 1** | 0.249 | 0.119 |
| **THRESHOLD 2** | 2.000 | 2.036 |
| **THRESHOLD 3** | 3.727 | 3.492 |
| **13 “I talked less than usual.”** |  |  |
| **THRESHOLD 1** | **0.449** | **0.446** |
| **THRESHOLD 2** | **1.806** | **1.445** |
| **THRESHOLD 3** | **2.811** | **2.461** |
| **14 “I felt lonely.”** |  |  |
| **THRESHOLD 1** | 0.834 | 0.897 |
| **THRESHOLD 2** | 2.356 | 2.027 |
| **THRESHOLD 3** | 3.013 | 2.852 |
| **15 “People were unfriendly.”** |  |  |
| **THRESHOLD 1** | 1.762 | 1.180 |
| **THRESHOLD 2** | 2.446 | 2.241 |
| **THRESHOLD 3** | 2.964 | 2.981 |
| **16 “I enjoyed life.”** |  |  |
| **THRESHOLD 1** | 0.574 | 0.495 |
| **THRESHOLD 2** | 1.849 | 1.938 |
| **THRESHOLD 3** | 3.661 | 3.168 |
| **17 “I had crying spells.”** |  |  |
| **THRESHOLD 1** | 1.027 | 1.697 |
| **THRESHOLD 2** | 2.402 | 2.622 |
| **THRESHOLD 3** | 2.964 | 3.456 |
| **18 “I felt sad.”** |  |  |
| **THRESHOLD 1** | 0.495 | 0.574 |
| **THRESHOLD 2** | 2.489 | 2.379 |
| **THRESHOLD 3** | 3.190 | 3.488 |
| **19 “I felt that people disliked me.”** |  |  |
| **THRESHOLD 1** | 1.760 | 1.187 |
| **THRESHOLD 2** | 2.492 | 2.361 |
| **THRESHOLD 3** | 2.930 | 2.982 |
| **20 “I could not get ‘going’.”** |  |  |
| **THRESHOLD 1** | **-0.040** | **0.427** |
| **THRESHOLD 2** | **1.528** | **1.762** |
| **THRESHOLD 3** | **2.427** | **2.777** |

Note: Values represent threshold values, which may be interpreted as relative level of depression required to endorse the item at each level (higher numbers represent higher severity of depression, with 0 interpretable as the average level of depression across the sample). Each item has three thresholds, representing the level of depression required to endorse “Some or a little of the time (1-2 days)”, “Occasionally or a moderate amount of the time (3-4 days)”, and “Most or all of the time (5-7 days)” respectively. Values are relative in nature and should only be interpreted in relation to each other. Bolded items represent somatic symptoms.
